# Relationships between alexithymia and food addiction: The Finnish version of Yale Food Addiction Scale and preliminary test of its psychometric properties

**DOI:** 10.3389/fpsyg.2023.1067872

**Published:** 2023-01-19

**Authors:** Ru Li, Jani Kajanoja, Jetro J. Tuulari, Linnea Karlsson, Hasse Karlsson, Max Karukivi

**Affiliations:** ^1^Department of Psychiatry, Turku University Hospital, University of Turku, Turku, Finland; ^2^Department of Clinical Medicine, FinnBrain Birth Cohort Study, Turku Brain and Mind Center, University of Turku, Turku, Finland; ^3^Department of Psychiatry, Satakunta Hospital District, Pori, Finland; ^4^Turku Collegium for Science, Medicine and Technology, TCSMT, University of Turku, Turku, Finland; ^5^Center for Population Health Research, Turku University Hospital, University of Turku, Turku, Finland; ^6^Department of Pediatrics and Adolescent Medicine, Turku University Hospital, University of Turku, Turku, Finland; ^7^Department of Adolescent Psychiatry, Turku University Hospital, University of Turku, Turku, Finland

**Keywords:** alexithymia, food addiction, Yale Food Addiction Scale, psychometric properties, factor analysis, structural equation modeling

## Abstract

**Background:**

It has long been suggested that addictive behaviors are associated with alexithymia, a personality trait characterized by difficulties in emotional awareness and expression. However, little is known about the role of alexithymia in food addiction.

**Objectives:**

The aim of this study was to investigate the relationship between alexithymia and food addiction. As part of the study, the validity of the Finnish version of Yale Food Addiction Scale (YFAS-F) was also investigated.

**Methods:**

The sample consisted of 360 parents from the FinnBrain Birth Cohort Study. The structural validity of the YFAS-F was evaluated by confirmatory factor analysis (CFA). Exploratory factor analysis (EFA) was used to explore the structure when proposed models were not supported by CFA. The associations of alexithymia as measured by the 20-item Toronto Alexithymia Scale and food addiction were examined using regression analyses followed by structural equation modeling.

**Results:**

Higher alexithymia was associated with more food addiction by conducting linear regression analysis (*B* = 0.013, *p* = 0.011) and structural equation modeling (*β* = 0.24, *p* < 0.001). Furthermore, a single-factor model for the 8 criteria of the YFAS-F was supported by CFA and showed acceptable internal reliability (KR-20 = 0.72), and a three-factor solution for the 20 items of the scale was suggested by EFA with good internal reliability (McDonald’s *ω* = 0.91 for the YFAS-F, 0.91 for component 1, 0.87 for component 2, and Spearman-Brown coefficient = 0.89 for component 3).

**Conclusion:**

The current study determined a significant relationship between alexithymia and food addiction, which suggests alexithymia as a relevant factor for food addiction and may provide clinical implications for interventions. Moreover, the YFAS-F appeared to be a valid and reliable tool to evaluate food addiction in our Finnish general population sample. Further studies on the psychometric properties of the YFAS-F in more diverse populations are recommended.

## 1. Introduction

The continually increasing prevalence of overweight and obesity is a serious health problem around the world and also in Finland (King, 2011; Vuorela et al., 2011). Unhealthy eating habits and taste preference for palatable foods have been viewed as risk factors contributing to obesity and overweight (Lanfer et al., 2012). Hyperpalatable foods, particularly ultra-processed foods with a high content of carbohydrates, sugar, fat and/or salt may possess addictive qualities, inducing strong craving and difficulties in controlling food intake in susceptible individuals (Gearhardt et al., 2011). With more understanding of the role of psychological factors and specific behaviors in the development of obesity, the concept of food addiction has gained increasing popularity among researchers in recent years (Penzenstadler et al., 2019). Regarding the measurement for food addiction, one of the most commonly used tool is the Yale Food Addiction Scale (YFAS). This is the first formal instrument to assess the addictive-like food intake originally designed based on the Diagnostic and Statistical Manual of Mental Disorders IV (DSM-IV-TR) diagnostic criteria for substance use disorders (Gearhardt et al., 2009). Several versions of the YFAS have been developed based on the original one for measuring food addiction in different contexts. For instance, a modified brief version of the YFAS (only one item assessing each symptom) and a child version of the YFAS have been proposed and applied (Flint et al., 2014). In addition, the YFAS has been translated into different language with sufficient validity and reliability in different countries, such as Chinese (Chen et al., 2015), French (Brunault et al., 2014), and Italian (Innamorati et al., 2015). However, there is no published research testing the psychometric properties of a Finnish version of the YFAS to measure food addiction in Finland.

Although there are still controversies about the terminology when referring to food addiction, mainly regarding whether to categorize it to substance-based or behavioral-based addictions, the presence of addictive-like eating is reasonably well established (Hebebrand et al., 2014; Gordon et al., 2018). For example, previous research has indicated that individuals with food addiction are characterized by food craving and a lack of control over their eating behavior (Meule, 2015). Moreover, findings from neurobiological studies have shown relations between intake of palatable foods (e.g., foods high in sugar and/or fat) and activity in the brain reward pathways (Grosshans et al., 2012). In addition, emotional functioning plays a role in addiction. For instance, emotion regulation difficulties are reportedly associated with substance use and addictive disorders, which may be attributed to problems in emotional stress regulation and inhibitory control related to frontal-limbic dysfunction (Li and Sinha, 2008; Tang et al., 2015).

Alexithymia, a personality trait characterized by difficulties in emotional awareness and expression, is a relevant factor in the context of addiction research. It was first proposed in 1973 with dimensions mainly involving difficulties in identifying and describing feelings, and externally oriented style of thinking (Sifneos, 1973). Alexithymia has long been reported to be associated with substance-related and addictive disorders such as alcohol use problems and pathological gambling (Parker et al., 2005; Kajanoja et al., 2019). It has been further suggested that alexithymia is linked to hypothalamic–pituitary–adrenal (HPA) axis dysfunction (Alkan et al., 2013), which may account for the role of alexithymia in addictions. Based on limited evidence, obese patients with food addiction seem to have more difficulties in emotion regulation and a higher level of alexithymia (El Archi et al., 2021). Nevertheless, research on associations between alexithymia and food addiction remains scarce.

The main aim of this study was to explore the relationship between alexithymia and food addiction, considering covariates including sample characteristics and psychological symptoms. As the psychometric properties of the Finnish YFAS (YFAS-F) are heretofore uninvestigated, we first intended to preliminarily validate the YFAS-F through determining the structural validity and internal consistency. Here, the YFAS-F was expected to be a valid and reliable tool for evaluating food addiction in our study sample of a Finnish general population. Furthermore, alexithymia was hypothesized to have associations with food addiction after taking into account potential confounders including sex and current psychological symptoms, and we also expected to establish a structural model confirming the link of alexithymia to food addiction.

## 2. Materials and methods

### 2.1. Study participants and procedure

This study is a cross-sectional sub-study based on the FinnBrain Birth Cohort Study^1^, a prospective cohort study exploring the impacts of prenatal and early life stress on child brain development and health (Karlsson et al., 2018). The families (*N* = 3,808) were recruited in Finland from maternal welfare clinics in the South-Western Hospital District and the Åland Islands during a verified pregnancy at the first trimester ultrasound performed at gestational week 12 between December 2011 and April 2015. The participants in the cohort have been followed up at 3-to 6-month intervals (the first 30 months) or 12-to 36-month intervals (from 36 months onwards) and the study is planned to continue for decades (more detailed information in Karlsson et al., 2018). 1,446 parents from the initial cohort were invited to the 5-year neuropsychological study visit, of which 415 parents responded to the YFAS-F. 55 respondents were excluded due to missing data on alexithymia questionnaire. Thus, the final sample for the present study consists of 360 parents.

The Ethics Committee of the Hospital District of Southwest Finland has approved the study protocol (14.6.2011 ETMK:57/180/2011 § 168). All procedures comply with the ethical standards of the national and institutional research committee on human experimentation, and with the 1964 Declaration of Helsinki as well as its later amendments.

### 2.2. Measures

#### 2.2.1. Food addiction

Food addiction was measured by the Yale Food Addiction Scale (YFAS). The YFAS is a self-report scale designed according to the DSM-IV-TR diagnostic criteria to evaluate the symptoms of food addiction experienced in the past 12 months (Gearhardt et al., 2009). The scale consists of 23 items for measuring 7 criteria and 2 items for clinically significant impairment or distress. Thus, the scale provides two scoring options, a continuous version indicating the “symptoms” of food addiction and a dichotomous version representing the “diagnosis” of food addiction. Food addiction “diagnosis” is met if three or more of the 7 criteria together with clinically significant impairment or distress are endorsed.

The translation of the original English YFAS into Finnish version was carried out by the research team, and back translation was carried out by an independent Finnish translator proficient in English. The final Finnish version (YFAS-F) was confirmed by the team after checking and discussing the accuracy of the translation and the discrepancies between the original and translated scales (see Supplementary material for comparisons of original version and Finnish translation of the scale).

#### 2.2.2. Alexithymia

Alexithymic traits were measured by the Toronto Alexithymia Scale (TAS-20), one of the most widely used self-report scales measuring alexithymia (Bagby et al., 1994; Joukamaa et al., 2001; Taylor et al., 2003). The scale consists of 20 items divided into 3 subscales assessing the dimensions including difficulty identifying feelings (DIF), difficulty describing feelings (DDF) and externally oriented thinking (EOT). The items are rated on a 5-point Likert scale that ranges from 1 (Strongly disagree) to 5 (Strongly agree), and thus a total score ranging from 20 to 100 is obtained. The Finnish translation of the TAS-20 was validated by Joukamaa et al. and showed satisfactory psychometric properties (Joukamaa et al., 2001; see Supplementary material).

#### 2.2.3. Psychological symptoms and sociodemographic

The Edinburgh Postnatal Depression Scale (EPDS), a valid and sensitive 10-item self-report questionnaire widely used for detecting postpartum depression among both mothers and fathers (Cox et al., 1987; Edmondson et al., 2010), was applied to assess depressive symptoms. The anxiety subscale of the Symptom Checklist-90 (SCL-90) was applied to assess anxiety symptoms experienced in the previous month (Derogatis et al., 1973; Holi et al., 1998). In the current sample, the Cronbach’s α was 0.87 and 0.88 for the EPDS and SCL-90, respectively.

According to the logistics of the birth cohort study, demographic data including age and sex were obtained during the first trimester of pregnancy. Participants’ self-reported height and weight were obtained at 4 years after childbirth to calculate body mass index (BMI). Information about education divided into three levels (Low: High school or lower; Mid: Vocational tertiary degree; High: University degree), economic satisfaction ranging from 0 to 15 (higher scores indicating more satisfaction), tobacco and alcohol use habits (nonregular/regular) were collected at 5 years after childbirth.

A brief summary of the measures of psychological symptoms, alexithymia and food addiction is presented in the Supplementary Table S1.

### 2.3. Statistical analysis

Descriptive statistics were presented as mean values (SD) and range since the data did not show severe violations of normality. Preliminary analyses included Student’s t-test and Chi-square test for group comparisons, and Spearman’s correlation coefficient for correlations between variables. After controlling for covariates selected based on the preliminary analyses, the associations between alexithymia and food addiction were examined by employing linear regression (for symptoms) and binary logistic regression analysis (for “diagnosis”). In order to maintain as large sample size as possible, individuals who missed background data (0.6% education, 0.8% alcohol use, and 29.2% BMI) were retained, and multiple imputation (MI) with predictive mean matching (PMM) as the model was applied to impute the missing values, producing 20 datasets as recommended for 10–30% missing information (Schafer, 1999; Graham et al., 2007). All the background variables were used as auxiliary variables in the imputation. The abovementioned analyses were performed using IBM SPSS 28.0.

Further, the link of alexithymia to food addiction was verified by conducting structural equation modeling (SEM), which is a confirmatory technique typically applied to test relationships between latent variables explained by observed variables (e.g., the dimensions of the TAS-20 and the symptoms of the YFAS-F in this study) by simultaneously estimating the overall fit of the measurement model and structural model (Ullman, 2006). Mplus 8.3 was used to conduct modeling.

Given that the original study on validating the YFAS suggested a single-factor structure for both the items and the criteria of the scale (Gearhardt et al., 2009), confirmatory factor analysis (CFA) was first conducted to test whether a single-factor model for the items fits our data. If the single-factor solution cannot be supported by CFA, exploratory factor analysis (EFA) with geomin (oblique) rotation was applied to examine the factorial structure of the YFAS-F and detect possible problematic items. The number of extracted components was determined according to the eigenvalues (greater than 1) and scree plot. Items with non-significant loading, loading value lower than 0.3, or cross-loading upon other components were excluded. In this study, the loading onto alternative factors above 0.30 with a difference smaller than 0.20 was suggested to be cross-loading (Howard, 2016). Furthermore, parallel analyses were performed to test the structural validity of the YFAS-F based on the 8 criteria (7 common symptoms and clinically significant impairment or distress). The internal consistency of the YFAS-F based on the remaining criteria was examined using Kuder–Richardson’s α (KR-20). For the internal consistency of the remaining items, McDonald’s omega was used for the scale and each component, whereas Spearman-Brown coefficient was used for 2-item component as suggested (Eisinga et al., 2013; Dunn et al., 2014).

Following indices were used to evaluate goodness-of-fit of the proposed models: normed Chi-square (χ^2^/degrees of freedom [df]), Root Mean Square Error of Approximation (RMSEA), Comparative Fit Index (CFI), Tucker-Lewis index (TLI), and Standardized Root Mean Residual (SRMR). The values of χ^2^/df < 3, RMSEA and SRMR <0.08, the probability of RMSEA ≤0.05 [P (RMSEA)] greater than 0.05 as well as CFI and TLI > 0.9 indicate a good model fit (Ullman, 2006). Given that the *χ^2^* statistic was highly sensitive to sample size as well as the multivariate normality assumption, its significance should not be the key index for model rejection and thus we did not use the result of *χ^2^* test for judging model fit (Schermelleh-Engel et al., 2003). Robust Maximum Likelihood (MLR) was used to estimate the parameters due to the non-normally distributed data. Statistical significance was set at *p* value <0.05 for all the tests.

## 3. Results

### 3.1. Sample characteristics and group comparisons by sex

Table 1 presents the descriptive statistics for variables and the proportion of missing data. The participants consist of 124 males and 236 females with their age ranging from 21 to 48 years old and an average BMI of 25.8 (SD = 5.2) kg/m^2^. Nearly half of the sample (*N* = 163, 45.3%) had received “High” education, and 79 (21.9%) reported “Low” education (High school or lower) and 116 (32.2%) “Mid” education (Vocational tertiary degree). 30 (8.3%) and 108 (30.0%) participants reported regular use of tobacco and alcohol, respectively. The data were suggested to be missing completely at random (MCAR) according to the Little’s MCAR test (*p* = 0.097), thus justifying MI method to handle missing data on background information.

**Table 1:** Descriptive statistics for variables and the proportion of missing data.

	*N* (%) or mean (SD), range	Missing %
Gender		–
*Male*	124 (34.4%)	
*Female*	236 (65.6%)	
Education		0.6%
*Low*	79 (21.9%)	
*Mid*	116 (32.2%)	
*High*	163 (45.3%)	
Tobacco use	30 (8.3%)	–
Alcohol use	108 (30.0%)	0.8%
FA diagnosis	18 (5.0%)	–
Age	32.5 (4.4), 21–48	–
ES	10.1 (3.3), 1–15	–
BMI (kg/m^2^)	25.8 (5.2), 17–54	29.2%
SCL-90	3.9 (5.0), 0–34	–
EPDS	4.6 (4.5), 0–22	–
TAS-20	41.6 (10.1), 22–73	–
FA symptoms	2.2 (0.9), 1–7	3.1%

Education: Low, high school or lower; Mid, vocational tertiary degree; High, university degree. ES, Economic satisfaction: from 0 (totally unsatisfied) to 15 (totally satisfied). BMI, body mass index. FA, food addiction. Diagnosis: based on the dichotomous score of the Finnish version of Yale Food Addiction Scale; Symptoms: based on the continuous score of the scale. EPDS, Edinburgh Postnatal Depression Scale; SCL-90, Symptom Checklist-90 (anxiety subscale). TAS-20, 20-item Toronto Alexithymia Scale. The pooled values after multiple imputation are presented when available.

According to the dichotomous score of the YFAS-F, 18 individuals (5.0%) met the diagnostic criteria for food addiction, most of which were females (N = 16). In terms of the endorsement rate of the YFAS-F criteria, the lowest rate was 2.2% for “Giving up social, occupational, or recreational activities,” and the highest rate was 46.9% for “Persistent desire or repeated unsuccessful attempts to quit.” There were 11 cases missing on the YFAS-F total score due to missing on individual item of the scale, but all of them reported no clinically significant impairment thus being classified into non-addiction group. The mean value of the YFAS-F symptom score was 2.2 (SD = 0.9) and median value was 2.0. No sex differences were observed in the level of food addiction symptoms (*p* = 0.541). In addition, the mean TAS-20 total score was 41.6 (SD = 10.1), with males reporting a higher level of alexithymia compared to females (43.6 vs. 40.5, *t* = 2.84, *p* = 0.005).

### 3.2. Structural validity and internal consistency of the YFAS-F

As most previous studies identified a single-factor structure of the original 22 items (excluding 3 item primers 17, 18 and 23), we run a single-factor model with using CFA at first. The fit indices to the data did not support the one-factor model for these items [*χ^2^* (209) = 1085.90, *p* < 0.001; RMSEA = 0.11, 90% CI: 0.10–0.11; CFI = 0.61; TLI = 0.57; SRMR = 0.11]. Item 24 showed a very low explained variance (*R*^2^ = 0.053) and was thus removed; however, the model fit did not improve after excluding this item.

**Figure 1:**
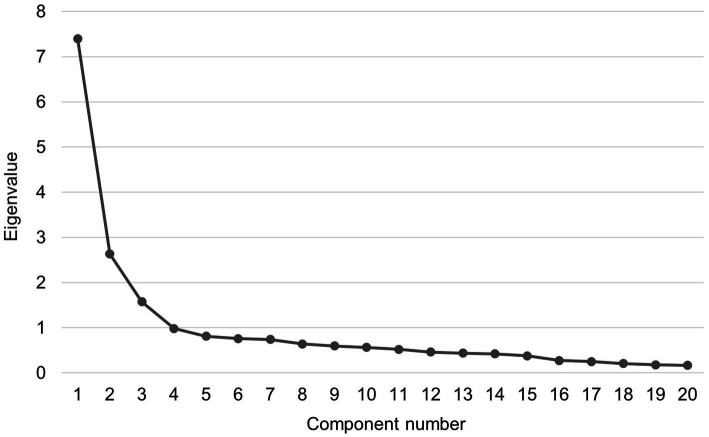
Scree plot of exploratory factor analysis for the 20-item Finnish version of Yale Food Addiction Scale.

EFA was then conducted to test the facets of the YFAS-F based on the items. The KMO value (0.90) and Bartlett’s test of sphericity (*χ*^2^ = 3952.39, *p* < 0.001) indicated that the data was suitable for factor analysis. Item 12 cross-loaded onto component 1 (loading value 0.41) and component 2 (loading value 0.40) thus being excluded. Both the scree plot (Figure 1) and eigenvalues suggested a 3-factor solution (eigenvalues for component 1 = 7.40, component 2 = 2.63, component 3 = 1.58) for the remaining 20 items. As presented in Table 2, most of the items formed the first component of food addiction, and five items and two items formed the components 2 and 3, respectively. The factor loadings for the 20 items in the 3-factor model ranged between 0.42 to 0.90. The McDonald’s ω was 0.91 for the 20-item YFAS-F, 0.91 for component 1, 0.87 for component 2, and Spearman-Brown coefficient was 0.89 for component 3, showing good internal consistency for these items.

**Table 2:** Descriptive statistics, component matrix, and factor loadings for items and criteria (bolded) of the Finnish version of Yale Food Addiction Scale.

Criteria and Items	Mean (SD)	Component and Factor Loadings		
		1	2	3
*(A) Substance taken in larger amount and for longer period than intended*		*0.51*		
Item 1	2.61 (1.06)	0.76		
Item 2	2.62 (1.06)	0.73		
Item 3	1.24 (0.62)	0.43		
*(B) Persistent desire or repeated unsuccessful attempts to quit*		*0.51*		
Item 4	2.03 (1.22)	0.82		
Item 22	0.46 (0.50)	0.63		
Item 24	0.40 (0.49)	-		
Item 25	1.55 (1.07)	0.63		
*(C) A great deal of time spent*		*0.68*		
Item 5	1.84 (0.96)	0.61		
Item 6	1.69 (1.04)	0.63		
Item 7	1.60 (0.85)	0.42		
*(D) Giving up social, occupational, or recreational activities*		*0.43*		
Item 8	1.07 (0.38)		0.79	
Item 9	1.04 (0.30)		0.93	
Item 10	1.03 (0.19)		0.85	
Item 11	1.06 (0.29)		0.63	
*(E) Continuing use despite physical or psychological problem*		*0.70*		
Item 19	0.14 (0.35)	0.56		
*(F) Tolerance*		*0.51*		
Item 20	0.03 (0.17)			0.85
Item 21	0.02 (0.16)			0.90
*(G) Withdrawal symptoms*		*0.74*		
Item 12	1.24 (0.61)	–		
Item 13	1.66 (0.97)	0.47		
Item 14	1.87 (1.02)	0.69		
(H) Clinically significant impairment or distress^*^		*0.71*		
Item 15	1.53 (0.99)	0.76		
Item 16	1.11 (0.47)		0.63	

^*^The loading of criterion H has a binomial distribution.

Regarding to the 8 criteria of the YFAS-F, the results of CFA demonstrated a sufficient fit of the single-factor model to the data used in our study [*χ^2^* (20) = 43.17, *p* = 0.002; RMSEA = 0.06, 90% CI: 0.03–0.08, P (RMSEA) = 0.29; CFI = 0.95; TLI = 0.93; SRMR = 0.04]. The factor loadings for the 8 criteria ranged between 0.43 and 0.74 (Table 2). The KR-20 coefficient was 0.72, suggesting acceptable internal consistency for the 8 criteria of the YFAS-F. The coefficient values did not significantly increase if any of these criteria were removed.

### 3.3. Associations between alexithymia and food addiction

According to the correlation matrix shown in Table 3, economic satisfaction, SCL-90 and EPDS scores, as well as TAS-20 total scores were significantly correlated to food addiction symptoms. In addition, the t-test showed associations of food addiction “diagnosis” with economic satisfaction (*p* = 0.004), SCL-90 and EPDS scores (*p* < 0.001), and BMI (*p* = 0.002). Due to a strong correlation between SCL-90 and EPDS scores, the SCL-90 score that had a relatively high correlation with food addiction was only retained as a covariate in subsequent analyses. The t-test and Chi-square test indicated that age, education, and tobacco and alcohol use habits were not significantly related to either YFAS-F symptoms or “diagnosis,” and thus they were excluded from further analyses.

**Table 3:** Spearman’s correlation matrix for continuous variables.

	1	2	3	4	5	6
1. Age						
2. ES	−0.06					
3. BMI (kg/m^2^)	0.16^*^	−0.13^*^				
4. SCL-90	−0.04	−0.14^**^	0.05			
5. EPDS	0.03	−0.22^**^	0.02	0.67^**^		
6. TAS-20	0.01	−0.13^**^	0.10	0.33^**^	0.41^**^	
7. FA symptoms	−0.01	−0.21^**^	0.11	0.21^**^	0.18^**^	0.18^**^

ES, Economic satisfaction: from 0 (totally unsatisfied) to 15 (totally satisfied). BMI, body mass index. FA symptoms: symptom levels of food addiction based on the continuous score of the Finnish version of Yale Food Addiction Scale. EPDS, Edinburgh Postnatal Depression Scale; SCL-90, Symptom Checklist-90 (anxiety subscale); TAS-20, 20-item Toronto Alexithymia Scale. ^*^p < 0.05, ^**^p < 0.01. The pooled values after multiple imputation are presented when available.

The t-test showed differences in the average TAS-20 scores between the food addiction and non-food addiction groups (41.2 vs. 49.3, *t* = −3.36, *p* < 0.001). After controlling for sex, economic satisfaction, BMI, and anxiety symptoms, the linear regression analysis revealed an association between the TAS-20 total score and the YFAS-F symptom score, while no relations between the TAS-20 total score and the YFAS-F “diagnosis” score were observed by using the logistic regression analysis (Table 4). However, when excluding anxiety symptoms from the logistic regression, the TAS-20 score was significantly related to the YFAS-F “diagnosis” score (OR = 1.07, 95% CI: 1.02–1.12, *p* = 0.006). In addition, we exploratively tested whether the relations between alexithymia and food addiction vary according to sex. The interaction between alexithymia and sex was not associated with the YFAS-F symptom (*p* = 0.785) or “diagnosis” score (*p* = 0.180), suggesting no sex differences in these associations.

**Table 4:** Associations between alexithymia and food addiction (linear regression for the symptoms and logistic regression for the “diagnosis”), controlling for selected covariates.

	*B* (95% CI)	*p*-value	OR (95% CI)	*p*-value
	*Outcome: FA Symptoms*		*Outcome: FA Diagnosis*	
Sex	0.18 (−0.00, 0.35)	0.052	4.79 (1.02, 22.56)	0.048
Economic satisfaction	−0.04 (−0.06, −0.01)	0.003	0.87 (0.75, 1.01)	0.075
SCL-90	0.04 (0.02, 0.06)	<0.001	1.08 (0.99, 1.18)	0.088
BMI (kg/m^2^)	0.02 (−0.00, 0.04)	0.054	1.09 (1.00, 1.18)	0.060
TAS-20 total scores	0.01 (0.01, 0.02)	0.002	1.04 (0.99, 1.10)	0.106

SCL-90, Symptom Checklist-90 (anxiety subscale); BMI, body mass index; TAS-20, 20-item Toronto Alexithymia Scale. FA Symptoms: symptom levels of food addiction based on the continuous score of the Finnish version of Yale Food Addiction Scale; FA Diagnosis: food addiction diagnosis based on the dichotomous score of the scale. B, unstandardized coefficients; OR, Odds Ratio; 95% CI, 95% confidence interval. The pooled values after multiple imputation are presented.

**Figure 2:**
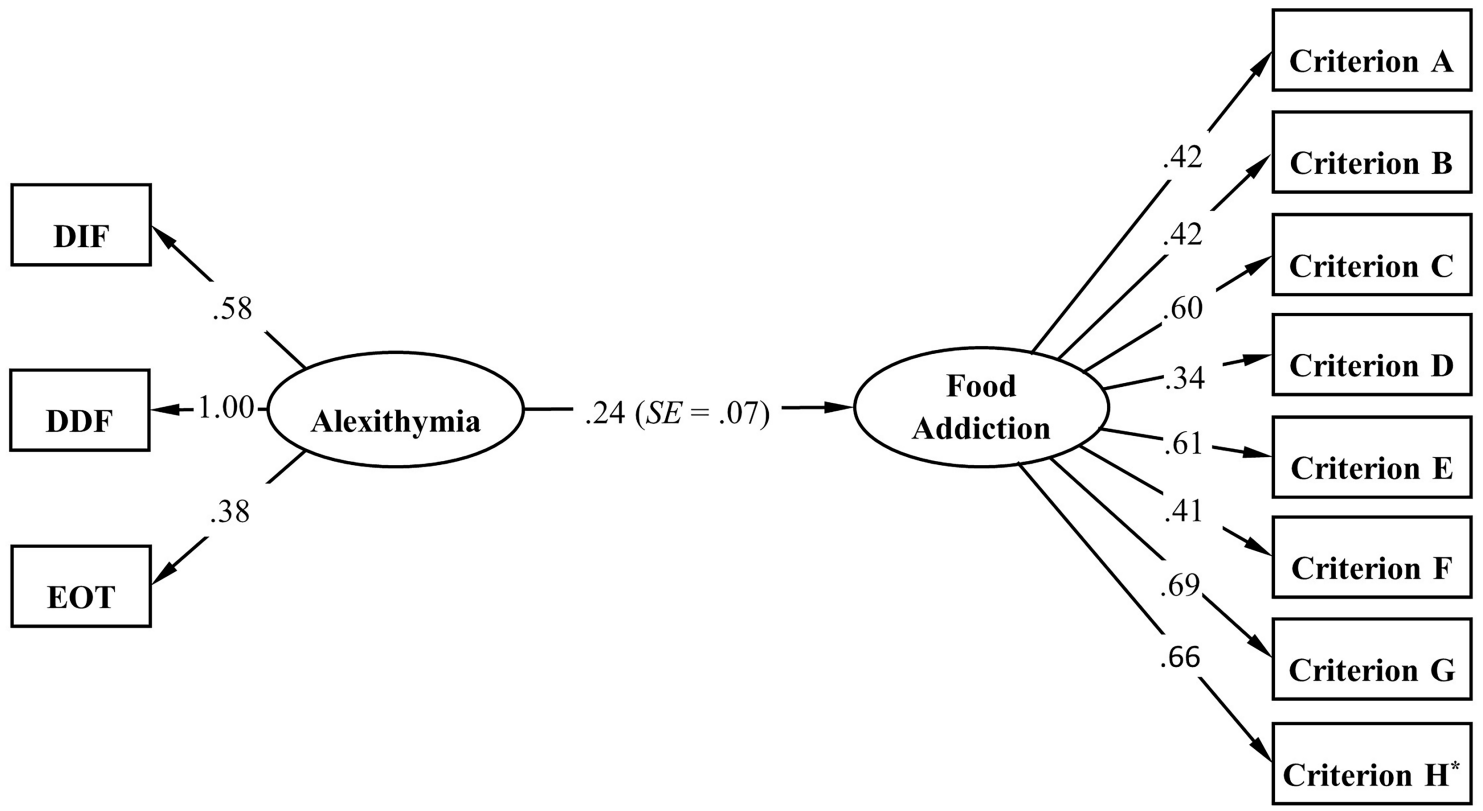
Standardized estimation results of SEM for the associations between alexithymia and food addiction. ^*^The loading of criterion H has a binomial distribution.

Further, SEM was conducted to confirm the relationship between alexithymia (measurement model with 3 dimensions as observed variables) and food addiction (measurement model with 8 criteria as observed variables). Likewise, CFA was first conducted to evaluate how the measurement model for alexithymia fits to our data. The Mplus output showed a negative residual variance of DDF but close to zero (−0.06) and therefore we fixed this parameter to zero in order to ensure correct modeling. The results showed a not perfect but adequate model fit [*χ^2^* (1) = 6.23, *p* = 0.013; RMSEA = 0.12, 90% CI: 0.05–0.22, P (RMSEA) = 0.061; CFI = 0.97; TLI = 0.92; SRMR = 0.03]. The RMSEA was higher than the suggested cut-off value, which is likely due to the single degree of freedom in this model. As RMSEA often falsely indicates a poor fit when a model has small degrees of freedom, it is suggested to be less important than other indices in such case (Kenny et al., 2015; Shi et al., 2022). In contrast, according to the CFI, TLI, and SRMR, the model demonstrated a reasonably sufficient fit to the data. As presented in Table 5, all the goodness-of-fit indices for SEM indicated a good model fit to the data. Figure 2 displays the structural equation model linking alexithymia to food addiction with the standardized estimation parameters. All the results presented in the model were statistically significant, and the model-based effect of alexithymia on food addiction was confirmed by SEM (*β* = 0.24, *SE* = 0.07, *p* < 0.001).

**Table 5:** Goodness-of-fit indices of structural equation modeling for alexithymia and food addiction.

Fit indices	Model values	Suggested values
*χ^2^* (df)	65.21 (44)	
*χ^2^*/df	1.48	<3
RMSEA [90% CI]	0.04 [0.02, 0.05]	<0.08
P (RMSEA)	0.89	>0.05
CFI	0.95	>0.90
TLI	0.93	>0.90
SRMR	0.06	<0.08

RMSEA, Root Mean Square Error of Approximation; P (RMSEA), Probability of RMSEA ≤ 0.05; 90% CI, 90% confidence interval; CFI, Comparative Fit Index; TLI, Tucker-Lewis index; SRMR, Standardized Root Mean Residual.

## 4. Discussion

The current study determined a significant association between alexithymia and food addiction. A higher level of alexithymia was related to more food addiction symptoms (controlling for the covariates including anxiety symptoms) and food addiction “diagnosis” (excluding anxiety symptoms). The structural relationship between alexithymia and food addiction was confirmed by conducting SEM. No sex differences were observed in their relationships. In addition, this study preliminarily validated the Finnish YFAS (YFAS-F) through testing the structural validity and internal consistency in general populations from a birth cohort.

The prevalence rate of food addiction in this study (5%) was lower than in other studies (around 10%) (e.g., Gearhardt et al., 2009; Brunault et al., 2014). However, the median symptom score was higher than the median scores reported in these studies, which indicated that the participants in our study may experience more food addiction symptoms but less clinical impairment or distress. In addition, although food addiction tends to be linked to obesity, it also occurs among normal-weight individuals (Pedram et al., 2013; Flint et al., 2014), which likely to some degree accounted for our finding that no correlations were found between BMI and food addiction.

Importantly, individuals with higher alexithymic traits experienced more food addiction symptoms, even after controlling for covariates including anxiety symptoms. The finding echoes previous evidence that alexithymia was associated with addictive behaviors such as pathological gambling (Parker et al., 2005). One of the potential mechanisms underlying the associations between alexithymia and addictions may be HPA axis dysfunction in alexithymic individuals, which reflects problematic regulation of stress responses (Alkan et al., 2013). Alexithymia was found to interact with perceived stress thus contributing to the development of stress-related mental health problems (Li et al., 2022). It is suggested that alexithymia may function as defense mechanism and addictive behaviors as maladaptive strategies to buffer psychological distress (Selfhout et al., 2009). Additionally, the proneness to addictions in alexithymic individuals can be speculated from the aspect of emotion regulation and social engagement. For instance, people may use addictive substances such as alcohol for alleviating their difficulties in emotional awareness and interpersonal function (Cruise and Becerra, 2018).

El Archi et al. (2021) have reported a higher alexithymia level in obese patients who were diagnosed as having food addiction, which is in line with the result of group comparison in our study. However, this association did not survive in the logistic regression analysis when adjusting for anxiety symptoms, suggesting that alexithymia had a relatively weak relation to the presence of food addiction compared to symptom levels of food addiction. Nevertheless, the results indicated that alexithymia still showed a trend toward the “diagnosis” score of the YFAS-F. In light of previous research showing that negative emotions were antecedents of binge eating (Dingemans et al., 2017), psychological distress such as anxiety may be involved in the link between alexithymia and food addiction. In addition, the relatively low detection rate (5%) in our sample may to some extent hinder detection of associations between alexithymia and “diagnosis” of food addiction. Notwithstanding, as there were no previous studies that investigated the link between alexithymia and food addiction considering the role of emotional distress, it is strongly recommended to consider the interaction effects of emotion factors when studying on the role of alexithymia in food addiction in future research.

According to previous studies, sex differences have been found in the prevalence of food addiction with mixed results (Pedram et al., 2013; Stojek et al., 2021). In our study, more females met the “diagnosis” of food addiction as measured by the YFAS-F compared to males, but no sex differences were observed in terms of the food addiction symptom levels. Moreover, the current results indicated no sex differences in the association between alexithymia and food addiction. Given that evidence on the role of alexithymia in food addiction is still lacking, more studies are needed to explore whether alexithymic traits as a developmental factor for food addiction varies across sex, which would improve understanding of food addiction and provide possibilities for personalized intervention.

By conducting SEM, we further confirmed the significant relationship between alexithymia and food addiction explained by the 3 dimensions of the TAS-20 and the 8 criteria of the YFAS-F with good overall fit of the measurement and structural model. To ensure correct modeling in the case of a small negative value of residual variance, a strategy reasonably used is to fix the residual variance of DDF to zero. We tried an *ad hoc* EFA and found that most of the items for DDF cross-loaded onto the other two distinct dimensions DIF and EOT, which is one important reason for leading to this phenomenon in our study. This is also in accordance with a previous study indicating that different types of alexithymia could be differentiated by either high DIF or EOT, but not DDF (Kajanoja et al., 2017). Overall, this is the first study determining their associations in general population with using SEM, a multivariate statistical method commonly applied for providing model-based causal evidence (Ullman, 2006). Our findings highlight the significance of alexithymia as a potential risk factor for food addiction, especially the symptom levels of food addiction, in a sample of Finnish general population.

Regarding the structural validity of the YFAS-F, in line with previous studies, a single-factor model for the criteria of the YFAS-F was supported by CFA with a sufficient model fit. However, a three-factor model for the items was identified in the present study, which is inconsistent with the original version of the YFAS (Gearhardt et al., 2009). Similarly, previous studies on the validation of the French and Italian versions of the YFAS have also shown a poor fit of the one-factor model for the items by using CFA or suggested a two-factor solution by EFA (Brunault et al., 2014; Innamorati et al., 2015). Considering differences in sample characteristics and the nature of the component, the three-dimension food addiction are explicable. For instance, the second dimension, consisting of four items measuring a criterion “Giving up social, occupational, or recreational activities” and item 16 (problems in the ability to function effectively such as daily routine, job/school, and social activities), represents social damage. The study by Brunault et al. (2014) suggested a two-factor model with high loading values of these items in the second component as well. Additionally, the third dimension consisted of two items (20 and 21) measuring the criterion “Tolerance,” which was reported by only 3.3% of the participants in our study. Unlike other substances such as tobacco and alcohol, the amount of daily food intake is relatively limited. Moreover, it is suggested that tolerance is not a central element in terms of behavioral addictions as increased specific behaviors are not for reducing negative emotions or increasing pleasure (Blaszczynski et al., 2008; Starcevic, 2016). Hence, the current results may to some extent reflect a behavioral-related feature of food addiction. Future research exploring potential subtypes of food addiction with a clinical sample would be beneficial to better understand its nature. In the present and previous studies, few items of the scale have shown low psychometric qualities, such as item 24 (Gearhardt et al., 2009; Meule et al., 2012; Chen et al., 2015). Hence, advanced approach such as Rasch modeling would be beneficial to the development of the instrument *via* detecting the properties of the items, improving the accuracy of measurements, and computing the performance of respondents (Boone, 2016).

Some limitations of this study should be acknowledged. Firstly, the YFAS-F was validated in the study sample of Finnish parents, and thus the generalization of the findings was limited by the sample characteristics. Secondly, regarding the validation of the YFAS-F, test–retest reliability and convergent/discriminant validity were not determined in the current study, which warrants future studies, ideally with concurrent measuring eating disorders such as binge eating and bulimia nervosa. Thirdly, due to the limited sample size, we did not randomly split the sample for EFA and CFA each or test the 3-factor solution for the items by using CFA in an additional group, which is worth considering in further studies. Fourthly, the YFAS 2.0 developed based on the DSM-5 criteria has not been translated into Finnish yet. Nevertheless, given the large overlap with the DSM-IV criteria and the reported less relevance of the added criteria in DSM-5 in the context of food, it is suggested that the YFAS can still be effectively used for screening and investigating food addiction (Meule and Gearhardt, 2014). The comparability of these two versions in Finnish population is worth exploring in the future. Lastly, although SEM suggested the model-based effect of alexithymia on food addiction, causal inferences cannot be made due to the use of cross-sectional data. Notwithstanding, given limited evidence in this regard, our findings provide insights into the role of alexithymia in food addiction. The current understanding would benefit from further follow-up investigation.

## 5. Conclusion

Individuals with a higher level of alexithymia experienced more food addiction symptoms, and no sex differences were observed in this relationship. Simultaneously, sufficient structural validity and internal reliability of the YFAS-F were determined, suggesting that this tool can be reasonably used in our sample. To the best of our knowledge, this is the first study examining the associations between alexithymia and food addiction using regression analysis and SEM among general population. Moreover, this is the first study testing the psychometric properties of the Finnish version of YFAS. The current findings suggest that alexithymia may play a role in food addiction, providing implications for implementing preventive and psychotherapeutic interventions such as emotion regulation training in individuals with food addiction. Given that this is only preliminarily validation and has some limitations regarding the generalizability beyond pregnancy cohort population, more studies are recommended to assess the overall validity of the YFAS-F in more diverse populations also in Finland.

## Data availability statement

The datasets presented in this article are not readily available because of restriction imposed by the Finnish law, and the study’s ethical permissions do not allow sharing of the data used in this study. Requests can be directed to the Board of the FinnBrain Birth Cohort Study. Requests to access the datasets should be directed to Hasse Karlsson, hasse.karlsson@utu.fi.

## Ethics statement

The studies involving human participants were reviewed and approved by the Ethics Committee of the Hospital District of Southwest Finland (14.6.2011 ETMK:57/180/2011 § 168). The patients/participants provided their written informed consent to participate in this study.

## Author contributions

RL: conceptualization, methodology, formal analysis, investigation, data curation, writing – original draft, and writing – review and editing. JK: conceptualization, supervision, methodology, investigation, data curation, and writing – review and editing. JT: investigation, data curation, and writing – review and editing. LK: investigation, data curation, project administration, resources, funding acquisition, and writing – review and editing. HK: investigation, data curation, project administration, resources, and writing – review and editing. MK: supervision, funding acquisition, methodology, and writing – review and editing. All authors contributed to the article and approved the submitted version.

## Funding

The work was supported by the Signe and Ane Gyllenberg Foundation and Academy of Finland. Funds for the open access publication fee were received from the State Research Grants of the Hospital District of Southwest Finland.

## Conflict of interest

The authors declare that the research was conducted in the absence of any commercial or financial relationships that could be construed as a potential conflict of interest.

## Publisher’s note

All claims expressed in this article are solely those of the authors and do not necessarily represent those of their affiliated organizations, or those of the publisher, the editors and the reviewers. Any product that may be evaluated in this article, or claim that may be made by its manufacturer, is not guaranteed or endorsed by the publisher.

## Footnotes

^1^ www.finnbrain.fi
