# Supplementary material for: Relationships between alexithymia and food addiction: The Finnish version of Yale Food Addiction Scale and preliminary test of its psychometric properties
Source: Front Psychol. 2023 Jan 19;14:1067872. doi: 10.3389/fpsyg.2023.1067872 (PMC9893275; doi:10.3389/fpsyg.2023.1067872)
Supplement: Supplementary file 1 [file Table_1.DOCX]

**Table S1.** Brief summary of the measures

| **Measures** | **Scores ranging** | **Objective** |
| --- | --- | --- |
| EPDS | 0–30 (Continuous) | Assess depressive symptoms in the past week |
| SCL-90 | 0–40 (Continuous) | Assess anxiety symptoms in the past month |
| TAS-20 total | 20–100 (Continuous) | Measure overall levels of alexithymic traits |
| *DIF* | 7–35 (Continuous) | Measure levels of difficulty identifying emotions |
| *DDF* | 5–25 (Continuous) | Measure levels of difficulty describing emotions |
| *EOT* | 8–40 (Continuous) | Measure the tendency of individuals to focus their attention externally |
| YFAS |  | Evaluate status of food addiction in the past year |
| *Symptoms* | 0–7 (Continuous) | Assess symptom count of food addiction |
| *Diagnosis* | 0/1 (Dichotomous) | For diagnosis of food addiction |

**Table S2. Yale Food Addiction Scale (YFAS): original version and Finnish translation**

| **Original** | **Finnish translation** |
| --- | --- |
| This survey asks about your eating habits in the past year. People sometimes have difficulty controlling their intake of certain foods such as:  - Sweets like ice cream, chocolate, doughnuts, cookies, cake, candy, ice cream  - Starches like white bread, rolls, pasta, and rice  - Salty snacks like chips, pretzels, and crackers  - Fatty foods like steak, bacon, hamburgers, cheeseburgers, pizza, and French fries  - Sugary drinks like soda pop  When the following questions ask about “certain foods” please think of any food similar to those listed in the food group or any other foods you have had a problem within the past year | Tässä kyselyssä selvitetään syömistottumuksiasi viimeisen vuoden aikana. Ihmisillä on joskus vaikeuksia hallita tiettyjen ruokien syömistä. Tällaisia ruokia ovat esimerkiksi:  - Makeat ruuat kuten suklaa, donitsit ja munkit, keksit, kakut, makeiset ja jäätelö  - Tärkkelyspitoiset ruuat kuten vaalea leipä, sämpylät, pasta ja riisi  - Suolaiset naposteltavat kuten sipsit, suolatikut ja suolakeksit  - Rasvaiset ruuat kuten pihvit, pekoni, jauhelihapihvit, hampurilaiset, pizza ja ranskalaiset perunat  - Sokeripitoiset juomat kuten limonadit  Kun seuraavissa väittämissä viitataan ‘tiettyihin ruokiin’, niin ajattele mitä tahansa yllä luetelluista ruuista tai muista ruuista, joiden kanssa sinulla on ollut hankaluuksia viimeisen vuoden aikana. |
| In the past 12 months: | Viimeisen 12 kuukauden aikana: |
| **Never (0)**  **Once a month (1)**  **2-4 times a month (2)**  **2-3 times a week (3)**  **4 or more times or daily (4)** | **Ei koskaan (0)**  **Kerran kuussa (1)**  **2-4 kertaa kuussa (2)**  **2-3 kertaa viikossa (3)**  **4 kertaa päivässä tai useammin (4)** |
| 1. I find that when I start eating certain foods, I end up eating much more than planned | 1. Kun alan syödä tiettyjä ruokia, syön enemmän kuin minun oli alun perin tarkoitus |
| 2. I find myself continuing to consume certain foods even though I am no longer hungry | 2. Jatkan tiettyjen ruokien syömistä vaikka en olisi enää nälkäinen. |
| 3. I eat to the point where I feel physically ill | 3. Syön niin pitkään kunnes alan voida pahoin. |
| 4. Not eating certain types of food or cutting down on certain types of food is something I worry about | 4. Olen huolissani siitä, miten voin välttää tai vähentää tiettyjen ruokien syömistä. |
| 5. I spend a lot of time feeling sluggish or fatigued from overeating | 5. Tunnen itseni usein veltoksi ja uupuneeksi koska olen syönyt liikaa. |
| 6. I find myself constantly eating certain foods throughout the day | 6. Huomaan, että syön jatkuvasti tiettyjä ruokia pitkin päivää. |
| 7. I find that when certain foods are not available, I will go out of my way to obtain them. For example, I will drive to the store to purchase certain foods even though I have other options available to me at home. | 7. Kun huomaan, että tiettyjä ruokia ei ole saatavilla, näen ylimääräistä vaivaa saadakseni niitä. Voin esimerkiksi lähteä käymään kaupassa ostamassa haluamiani ruokia, vaikka minulla olisi kotona muutakin syötävää. |
| 8. There have been times when I consumed certain foods so often or in such large quantities that I started to eat food instead of working, spending time with my family or friends, or engaging in other important activities or recreational activities I enjoy. | 8. Olen joskus syönyt niin suuria määriä ruokaa, että syöminen on syrjäyttänyt muita tärkeitä asioita kuten työskentelemisen, perheen ja ystävien kanssa olemisen tai muita tärkeitä asioita tai harrastuksia, joista pidän. |
| 9. There have been times when I consumed certain foods so often or in such large quantities that I spent time dealing with negative feelings from overeating instead of working, spending time with my family or friends, or engaging in other important activities or recreational activities I enjoy. | 9. Olen joskus syönyt niin suuria määriä ruokaa, että syömiseen liittyvien kielteisten tunteiden käsitteleminen on syrjäyttänyt muita tärkeitä asioita kuten töissä käymisen, perheen ja ystävien kanssa olemisen tai muita tärkeitä asioita tai harrastuksia, joista pidän. |
| 10. There have been times when I avoided professional or social situations where certain foods were available, because I was afraid I would overeat. | 10. Olen joskus vältellyt sellaisia työhön tai vapaa-aikaan liittyviä tilaisuuksia, joissa on ruokaa tarjolla, koska pelkään että saatan syödä liikaa. |
| 11. There have been times when I avoided professional or social situations because I was not able to consume certain foods there. | 11. Olen joskus vältellyt sellaisia työhön tai vapaa-aikaan liittyviä tilaisuuksia, joissa on ruokaa tarjolla, koska pelkään, että en voi syödä jotain niissä tarjottavia ruokia. |
| 12. I have had withdrawal symptoms such as agitation, anxiety, or other physical symptoms when I cut down or stopped eating certain foods. (Please do NOT include withdrawal symptoms caused by cutting down on caffeinated beverages such as soda pop, coffee, tea, energy drinks, etc.) | 12. Kun olen vähentänyt tai lopettanut tiettyjen ruokien syömisen, olen saanut vieroitusoireita kuten ärtyisyyttä, ahdistuneisuutta tai muita fyysisiä oireita (älä kuitenkaan laske mukaan kofeiinipitoisten juomien kuten kahvin tai energiajuomien käytön lopettamiseen tai vähentämiseen liittyviä oireita). |
| 13. I have consumed certain foods to prevent feelings of anxiety, agitation, or other physical symptoms that were developing. (Please do NOT include consumption of caffeinated beverages such as soda pop, coffee, tea, energy drinks, etc.) | 13. Olen syönyt tiettyjä ruokia hillitäkseni esimerkiksi ärsyyntyneisyyttä, ahdistuneisuutta tai muita fyysisiä oireita (älä kuitenkaan laske mukaan kofeiinipitoisten juomien kuten kahvin tai energiajuomien nauttimista). |
| 14. I have found that I have elevated desire for or urges to consume certain foods when I cut down or stop eating them. | 14. Kun yritän vähentää tai lopettaa tiettyjen ruokien syömistä, huomaan himoitsevani niitä entistä enemmän. |
| 15. My behavior with respect to food and eating causes significant distress. | 15. Syömiseen ja ruokaan liittyvä käytökseni huolestuttaa minua. |
| 16. I experience significant problems in my ability to function effectively (daily routine, job/school, social activities, family activities, health difficulties) because of food and eating. | 16. Minulla on ruuan ja syömisen johdosta merkittäviä vaikeuksia toimia tehokkaasti arkipäivän elämässä (kuten päivärutiineissa, työssä tai koulussa, sosiaalisessa elämässä perheen ja ystävien kanssa). |
|  |  |
| **No/Yes (0/1)** | **Ei/ Kyllä (0/1)** |
| 17. My food consumption has caused significant psychological problems such as depression, anxiety, self-loathing, or guilt. | 17. Syöminen on aiheuttanut minulle henkisiä ongelmia kuten masentuneisuutta, ahdistuneisuutta, itseinhoa tai syyllisyyttä. |
| 18. My food consumption has caused significant physical problems or made a physical problem worse. | 18. Syöminen on aiheuttanut minulle merkittäviä fyysisiä vaivoja tai pahentanut olemassa olevia vaivojani. |
| 19. I kept consuming the same types of food or the same amount of food even though I was having emotional and/or physical problems. | 19. Jatkan tiettyjen ruokien syömistä tai syön yhtä paljon kuin ennenkin, vaikka se aiheuttaa minulle fyysisiä tai henkisiä vaivoja. |
| 20. Over time, I have found that I need to eat more and more to get the feeling I want, such as reduced negative emotions or increased pleasure. | 20. Olen ajan mittaan huomannut, että minun pitää syödä entistä enemmän, jotta syöminen aiheuttaisi haluamiani tuntemuksia, kuten mielihyvää tai kielteisten tunteiden vähenemistä. |
| 21. I have found that eating the same amount of food does not reduce my negative emotions or increase pleasurable feelings the way it used to. | 21. Olen huomannut, että saman ruokamäärän syöminen ei enää vähennä kielteisiä tunteita tai aiheuta myönteisiä tunteita samalla tavalla kuin aikaisemmin. |
| 22. I want to cut down or stop eating certain kinds of food. | 22. Haluaisin vähentää tai lopettaa kokonaan tiettyjen ruokien syömisen. |
| 23. I have tried to cut down or stop eating certain kinds of food. | 23. Olen yrittänyt vähentää tai lopettaa kokonaan tiettyjen ruokien syömisen. |
| 24. I have been successful at cutting down or not eating these kinds of food | 24. Olen onnistunut vähentämään tai lopettamaan näiden ruokien syömisen. |
|  |  |
| **1 time, 2 times, 3 times, 4 times, 5 or more times** | **Kerran, 2 kertaa, 3 kertaa, 4 kertaa, 5 kertaa tai useammin** |
| 25. How many times in the past year did you try to cut down or stop eating certain foods altogether? | Kuinka monta kertaa olet viimeisen vuoden aikana koittanut lopettaa tiettyjen ruokien syömistä kokonaan? |

Note: #17, #18, #23 are NOT scored but are primers for other questions.

Scoring:

The following cut-offs were developed for the continuous questions, 0 = criterion not met, 1 = criterion is met:

The following questions are scored 0 = (0), 1 = (1): #19, #20, #21, #22

The following question is scored 0 = (1), 1 = (0): #24

The following questions are scored 0 = (0 thru 1), 1 = (2 thru 4): #8, #10, #11

The following questions are scored 0 = (0 thru 2), 1 = (3 & 4): #3, #5, #7, #9, #12, #13, #14, #15, #16

The following questions are scored 0 = (0 thru 3), 1 = (4): #1, #2, #4, #6

The following questions are scored 0 = (0 thru 4), 1 = (5): #25

After computing cut-offs, sum up the questions under each substance dependence criterion (Tolerance, Withdrawal, Clinical Significance, etc.). If the score for the criterion is ≥ 1, then the criterion has been met and is scored as 1. If the score = 0, then the criteria has not been met.

To score the continuous version of the scale, which resembles a symptom count without diagnosis, add up all of the scores for each of the criterion (e.g., Tolerance, Withdrawal, Use Despite Negative Consequence). Do not add clinical significance to the score. This score should range from 0 to 7.

To score the dichotomous version, which resembles a diagnosis of substance dependence, compute a variable in which clinical significance must = 1 (items 15 or 16 =1), and the symptom count must be > 3. This should be either a 0 or 1 score (no diagnosis or diagnosis met.)

**Table S3. 20-item Toronto Alexithymia Scale (TAS-20): original version and Finnish translation**

| **Original** | **Finnish translation** |
| --- | --- |
| **1 = Strongly disagree**  **2 = Disagree**  **3 = Neither disagree nor agree**  **4 = Agree**  **5 = Strongly agree** | **1 = Ei lainkaan pidä paikkansa**  **2 = Ei juuri pidä paikkansa**  **3 = En osaa sanoa**  **4 = Pitää melkolailla paikkansa**  **5 = Pitää täysin paikkansa** |
| 1. I am often confused about what emotion I am feeling | 1. Olen usein epävarma siitä, mitä milloinkin tunnen. |
| 2. It is difficult for me to find the right words for my feelings | 2. Minun on vaikea löytää oikeita sanoja kuvatakseni tunteitani. |
| 3. I have physical sensations that even doctors don’t understand | 3. Minulla on fyysisiä tuntemuksia, joita lääkäritkään eivät ymmärrä. |
| 4. I am able to describe my feelings easily | 4. Minun on helppo kuvailla tunteitani. |
| 5. I prefer to analyze problems rather than just describe them | 5. Mieluummin erittelen ja tutkin ongelmia kuin vain kuvailen niitä |
| 6. When I am upset‚ I don’t know if I am sad‚ frightened‚ or angry | 6. Kun olen poissa tolaltani, en tiedä olenko surullinen, peloissani vai vihainen. |
| 7. I am often puzzled by sensations in my body | 7. Olen usein ymmälläni kehoni tuntemuksista. |
| 8. I prefer to just let things happen rather than to understand why they turned out that way | 8. Annan mieluummin asioiden mennä omalla painollaan kuin mietin, mistä ne oikein johtuvat. |
| 9. I have feelings that I can’t quite identify | 9. Minulla on tunteita, joita en täysin pysty tunnistamaan. |
| 10. Being in touch with emotions is essential (R) | 10. On erityisen tärkeää olla kosketuksissa tunteisiinsa. |
| 11. I find it hard to describe how I feel about people | 11. Minun on vaikea kuvailla tunteita, joita toiset ihmiset minussa herättävät. |
| 12. People tell me to describe my feelings more | 12. Ihmiset ovat kehottaneet minua kertomaan enemmän tunteistani. |
| 13. I don’t know what’s going on inside me | 13. En tiedä, mitä sisimmässäni oikein tapahtuu. |
| 14. I often don’t know why I am angry | 14. En aina tiedä, miksi olen vihainen. |
| 15. I prefer talking to people about their daily activities rather than their feelings | 15. Mieluummin puhun ihmisten kanssa heidän päivittäisistä puuhistaan kuin heidän tunteistaan. |
| 16. I prefer to watch “light” entertainment shows rather than psychological dramas | 16. Katselen mieluummin kevyttä viihdettä kuin psykologisia ohjelmia. |
| 17. It’s difficult for me to reveal my innermost feelings‚ even to close friends | 17. Minun on vaikea paljastaa sisimpiä tuntojani edes läheisille ystävilleni. |
| 18. I can feel close to someone‚ even in moments of silence (R) | 18. Voin tuntea läheisyyttä toiseen ihmiseen, vaikka oltaisiin hiljaa. |
| 19. I find examination of my feelings useful in solving personal problems (R) | 19. Olen huomannut, että omien tunteiden kuunteleminen ja pohtiminen auttaa henkilökohtaisten ongelmien ratkaisemisessa. |
| 20. Looking for hidden meanings in movies or plays distracts from their enjoyment | 20. Elokuvista tai näytelmistä häviää nautinto, jos niistä yrittää etsiä syvällisiä merkityksiä |

Note:

The TAS-20 has 3 subscales:

• Difficulty Identifying Feeling (DIF) subscale is used to measure difficulty identifying emotions. 7 items – 1, 3, 6, 7, 9, 13, 14.

• Difficulty Describing Feelings (DDF) subscale is used to measure difficulty describing emotions. 5 items – 2, 4, 11, 12, 17.

• Externally Oriented Thinking (EOT) subscale is used to measure the tendency of individuals to focus their attention externally. 8 items – 5, 8, 10, 15, 16, 18, 19, 20.
